# Experiencing loneliness in parenthood: a scoping review

**DOI:** 10.1177/17579139211018243

**Published:** 2021-07-21

**Authors:** R Nowland, G Thomson, L McNally, T Smith, K Whittaker

**Affiliations:** School of Community Health and Midwifery, University of Central Lancashire Brooke Building, Preston PR2 1HE, UK; University of Central Lancashire, Preston, UK; University of Central Lancashire, Preston, UK; University of Central Lancashire, Preston, UK; University of Central Lancashire, Preston, UK

**Keywords:** parental, mother, father, loneliness, parenthood, perceived social isolation

## Abstract

**Aims:**

Chronic loneliness is experienced by around a third of parents, but there is no comprehensive review into how, why and which parents experience loneliness. This scoping review aimed to provide insight into what is already known about parental loneliness and give directions for further applied and methodological research.

**Methods:**

Searches for peer-reviewed articles were undertaken in six databases: PsycINFO, Medline, CINAHL, Embase, Web of Science and Scopus, during May 2019 to February 2020. We searched for English studies which examined loneliness experienced during parenthood, including studies that involved parents with children under 16 years and living at home and excluding studies on pregnancy, childbirth or postbirth hospital care.

**Results:**

From 2566 studies retrieved, 133 were included for analysis. Most studies (*n* = 80) examined the experience of loneliness in specific groups of parents, for example, teenage parents, parents of a disabled child. Other studies examined theoretical issues (*n* = 6) or health and wellbeing impacts on parents (*n* = 16) and their offspring (*n* = 17). There were 14 intervention studies with parents that measured loneliness as an outcome. Insights indicate that parental loneliness may be different to loneliness experienced in other cohorts. There is evidence that parental loneliness has direct and intergenerational impacts on parent and child mental health. Some parents (e.g. with children with chronic illness or disability, immigrant or ethnic minority parents) also appear to be at increased risk of loneliness although evidence is not conclusive.

**Conclusion:**

This work has identified key gaps with further international, comparative and conceptual research needed.

## Introduction

Loneliness is now widely understood as a painful subjective experience when the social connections a person has do not meet their interpersonal needs in respect to quality of or quantity of friendship or social contact.^1^ Loneliness can be experienced in the presence of others and is different from objective measures of social connection, such as social isolation (the absence of social relationships) and social network size (number of social connections).^2^

Much of the existing loneliness literature has been conducted with undergraduate and elderly populations and shows that loneliness has associations with poor mental and physical health,^3,4^ impacting on early mortality.^5^ This focus in the literature means that interventions for loneliness are based on knowledge about the experience of loneliness limited to these restricted populations. It is therefore not known whether and how the experience of loneliness differs in other populations.

One such population where there has been little examination of the experience of loneliness is parents. Surveys have shown that around a third of parents in the UK report experiencing loneliness often or always^6^ and research studies have shown similar prevalence, with 30% of parents experiencing high and persistent levels of loneliness over time.^7^ However, despite such high numbers of parents being affected, there is currently no comprehensive synthesis of existing knowledge on the impacts and experiences of loneliness in this population and no reviews in this area. Given the mental and physical health impacts of loneliness in other populations,^3–5^ it is important to establish what is known about the health implications of loneliness in parenthood and whether there is evidence of intergenerational effects, impacting health and wellbeing of their offspring. Establishing what is known about the experiences of loneliness and which parents are at an increased risk of experiencing loneliness is important to underpin and direct appropriate strategies, support and future research.

### The current study

We aimed to address the current knowledge gap by undertaking a scoping review to map existing research evidence on parental loneliness, to establish what is already known about experiences and impacts of loneliness in parenthood, and which parents are at increased risk of experiencing loneliness. As we aimed to examine evidence from disparate or heterogeneous sources, rather than seeking only the best evidence to answer a specific question, a scoping review methodology was considered appropriate.^7^ This methodology enables an examination and synthesis of the extent, range and nature of research on parental loneliness, to inform future systematic reviews, and to identify gaps in the literature.^8^ In the current scoping review, we focused specifically on loneliness, rather than other measures of social connection (i.e. social support, social isolation), in order to establish what is known about parental loneliness and what research has been conducted in this specific area.

## Method

### Search strategy

We conducted some preliminary scoping searches during October 2018 to January 2019 which identified the diversity of study types and findings in this research area and informed our search strategy, review protocol and choice of review type. We used the scoping review stages outlined by Arksey and O’Malley^8^ and Levac et al.^9^ as a framework for the review. The following search terms were developed: (mother* or maternal or parent* or father* or paternal) AND (lonel* or ‘perceived social isolat*’). The search strategy was adapted to meet the truncation and Boolean operations of each database as appropriate (see Supplemental Information 1). Initial database searches were conducted in May 2019 and repeated in February 2020 in six bibliographic databases: PsycINFO, Medline, CINAHL, Embase, Web of Science and Scopus. Handsearching was also conducted, involving reference list searching of reviews and key papers and google scholar searches (first 200 hits for search terms).

### Inclusion and exclusion criteria

Included studies were those that examined the following: (1) prevalence and/or experiences of loneliness for mothers and fathers, (2) impacts of parental loneliness on mothers’ and fathers’ health and wellbeing and relationships with their child/ren, and (3) the impacts of parental loneliness on the child, including intergenerational transmission of loneliness. Inclusion and exclusion criteria are detailed in Table 1. We only included studies involving parents with children under 16 years old and living at home, thereby capturing insights with parents who had full parental accountabilities and responsibilities. All study types were included, but we excluded grey literature such as books and book chapters, dissertations, editorials, opinion pieces, commentaries, book or movie reviews, and erratum. There was no date restriction on searches, but only studies written in English were included. Systematic/literature reviews undertaken into parental loneliness were not included in our synthesis and mapping, but we reported on the numbers of relevant reviews identified in this area.

**Table 1:** Inclusion and exclusion criteria

	Inclusion	Exclusion	Search terms
Population	Mothers, fathers, (biological or step parents), children 16 years and under and living in the family home	Non-parental caregivers (e.g. grandparents), pregnant women, adoptive/foster parents Mothers, fathers (biological or step parents with children over the age of 16 and/or not living in the family home)	mother* or maternal or parent* or father* or paternal
Exposure	Loneliness, perceived social isolation	Other mental health issues (e.g. depression) but do not explicitly refer to loneliness	Lonel* or ‘perceived social isolat*’
Outcome	Experiences, attitudes and opinions of loneliness, prevalence of loneliness, impacts of parental loneliness on parent or child’s health and wellbeing	Studies that examine loneliness in child only, pregnancy, birth experiences	
Study types	All research study design	Books and book chapters, editorials, erratum, opinion pieces, conference abstracts, reviews, dissertations, protocols	
Language	English only	Non-English	

### Screening

Papers identified from database searches were downloaded to Endnote and duplicates removed. Title and abstract screening were conducted in Rayyan.^10^ One reviewer independently screened titles and abstracts for eligibility, with a sample of 20% of the papers screened by the rest of the team to check for accuracy prior to independent screening. Papers selected for full-text screening were then sourced and examined by one author independently, noting decision-making and reasons for exclusion. A sample of 50% of full-text papers were screened by at least one other reviewer prior to independent screening. Percentage of agreement for title and abstract screening was 93.2% and 88.73% for full-text screening. Agreement was made by consensus, with disagreements resolved through discussion. It is becoming widely accepted that double screening all papers in a systematic review is more appropriate to reduce articles missed due to human error.^11,12^ However, where reviews are conducted by experienced reviewers missing studies have been shown to have negligible or no impact on meta-analysis findings.^13^ Thus, double screening 20% of title and abstract (where there was higher agreement) and 50% of full-text screening was deemed appropriate for this scoping review following reconciliation exercises^11^ because it was an experienced review team.

### Data extraction and synthesis

Data were extracted from all selected texts using a data extraction sheet developed by the authors, with at least 20% of data extracted charted by two authors independently.^14^ Once sufficient agreement (>80%) was reached in the trial phase, the first author independently applied the tool to the remaining studies. During data extraction, review team meetings were held periodically to ensure accuracy of data extraction and to discuss any anomalies. Studies were assigned categories in discussion with the full review team. For each of these categories, we collated the key information and summaries of findings and then conducted a narrative synthesis. We did not conduct a meta-analysis because the purposes of the scoping review were to map and synthesis literature on a wide topic, involving disparate methodologies and measures and due to the lack of homogeneity such an analysis was not deemed appropriate.

## Results

**Figure 1:**
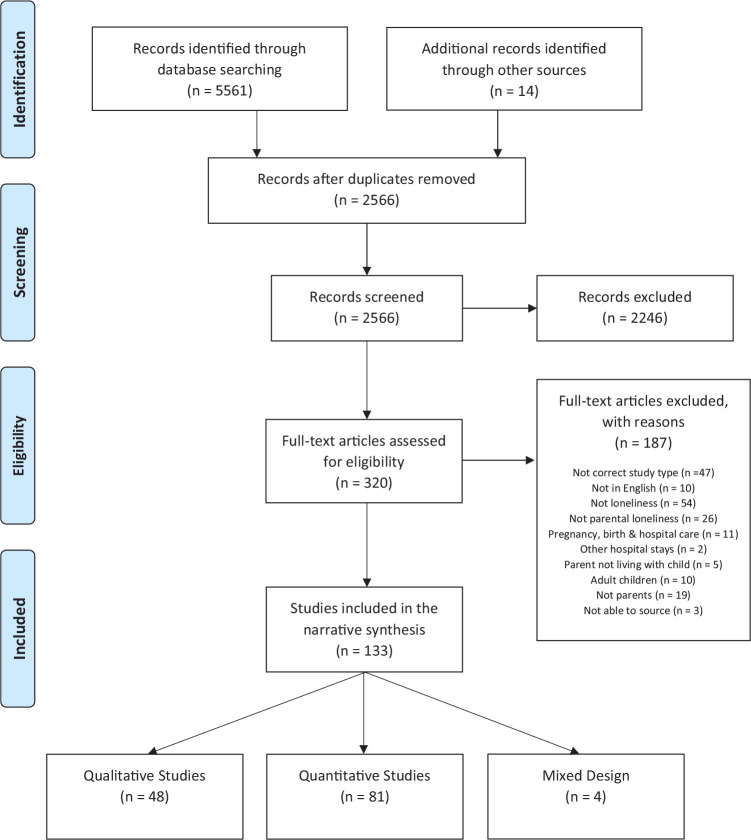
PRISMA flow diagram

A total of 133 studies were included. The PRISMA diagram outlines the results of the systematic searches and screening (Figure 1), and Supplemental Table 2 (see Supplemental material) provides a description of the included studies. Only two review papers were identified, both narrative reviews focusing on loneliness within the family unit (i.e. in relation to marital or family conflict) and impacts on the child,^15,16^ rather than focusing specifically on loneliness experienced in parenthood.

**Table 2:** Studies examining impacts of parental loneliness on child’s mental health and social competence

Author	Year	Country	Child’s age	Design	Loneliness measure	Findings
Alvik^72^	2014	Norway	6 months	Long	Single item measure	Mothers’ loneliness at 30 weeks in pregnancy predicted child’s low scores on problem-solving aspect of Ages and Stages Questionnaire at 6 months
Al-Yagon^73^	2007	Israel	9–10 years	CS	ESL (mothers), LSDQ (child)	Mother’s loneliness associated with child’s internalising behaviours (not child’s loneliness), but when maternal resources included in analysis, mothers’ loneliness did not predict any child measures
Henwood and Solano^74^	1994	US	6–7 years	CS	ABLS (parents), LSDQ (child)	Association between mothers and child’s loneliness, but not between fathers and their child’s loneliness
Junttila and Vauras^75^	2009	Finland	10–11 years	Long	UCLA^21^ (parents), PNDLS (child)	Mother’s and father’s loneliness predicted peer-evaluated cooperating skills of girls (but not boys), which predicted their social and emotional loneliness
Junttila et al.^76^	2007	Finland	10–11 years	CS	UCLA (parents) PNDLS (child)	Association between high parental loneliness and low parenting self-efficacy. Parenting self-efficacy was related to children’s loneliness
Luoma et al.^68^	2019	Finland	16–17 years	Long	Single item, ‘Do you feel lonely?’	Mother’s prenatal loneliness predicted the child’s internalising problems in adolescence
Salo et al.^77^	2020	Turkey	10–11 years	Long	UCLA (parents), PNDLS (child)	Long-term loneliness of sons was predicted by their father’s loneliness and daughters by mothers
Stednitz and Epkins^78^	2006	US	9–12 years	CS	SELSA	Mother’s loneliness predicted girls’ self-reported social anxiety
Zafar Kausar^79^	2015	India	13–17 years	CS	UCLA	Mothers’ high loneliness predicted adolescent’s lower social competence, hostility and fear of negative evaluation

SELSA = Social and Emotional Scale for Adults^80^; SELSA-S = Social and Emotional Loneliness Scale for Adults^81^; PNDLS = Peer Network and Dyadic Loneliness Scale^82^; ABLS = Abbreviated Loneliness Scale^83^; ESL = Emotional and Social Loneliness^84^; LSDQ = Loneliness and Social Dissatisfaction Questionnaire.^85^

Most of the included studies were conducted in America (*n* = 46; 34.59%) and Canada (*n* = 13; 9.77%), with others conducted in Australia (*n* = 9; 6.77%), Finland (*n* = 8; 6.02%), Sweden (*n* = 7; 5.26%), Netherlands (*n* = 7; 5.26%), Israel (*n* = 7; 5.26%) and England (*n* = 7; 5.26%). The included studies had publication dates from 1974 to 2020, with around half (*n* = 66; 49.62%) published in the last 10 years and 30.83% (*n* = 41) in the last 5 years. All bar one of the included studies were published as peer-reviewed journal articles; with the remaining study published as a short report.^17^ Most studies used a quantitative design (*n* = 81; 60.90%), with the rest using either a qualitative (*n* = 48; 36.09%) or mixed methods (*n* = 4; 3.01%) design. Most studies examined loneliness in mothers only (*n* = 90; 67.67%), with others exploring relationships in both parents (*n* = 39; 29.32%). Only three studies examined loneliness in fathers only, with one exploring the experience of living with a partner with postnatal depression rather than fathers’ loneliness during parenthood.^18^ One study examined loneliness in transgender men^19^ and the other in gender variant parents.^20^ Most studies were cross-sectional (*n* = 102; 76.69%), with only 31 (23.31%) using a longitudinal design. More than half of the studies that used a quantitative or mixed design (*n* = 78, 91.76%) used a loneliness scale, such as the UCLA loneliness measure^21^ (*n* = 40; 47.06%), but with varying versions (i.e. number of items). Eleven (12.94%) of the quantitative studies used single item measures of loneliness, but the questions and response items varied. In quantitative or mixed design studies where a loneliness scale was not used (*n* = 6, 4.51%), parents were asked to detail any problems they were experiencing via open text answers or preselected responses including loneliness (i.e. frequency counts typically reported).

### Data analysis

**Figure 2:**
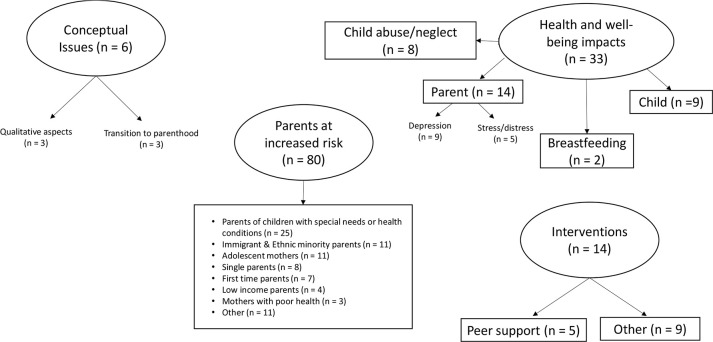
Category mapping of studies on parental loneliness Other category includes sub-categories where there are two or less studies, which includes housing (*n* = 2), partner violence/abuse (*n* = 2), military wives (*n* = 1), specific work patterns (*n* = 2), parents with substance abuse (*n* = 2) and gender variant parents (*n* = 2).

The categories of the included studies are outlined in Figure 2 and described below.

#### *Theoretical aspects of loneliness in parenthood (n* *=* *6)*

Only six studies examined theoretical issues relating to loneliness in parenthood. Three of these studies examined changes in loneliness associated with becoming a parent. One used a longitudinal design and found loneliness to be stable across pregnancy, infant and toddler years in mothers and fathers.^22^ Another study found no changes in women’s wellbeing, but men who became fathers became lonelier, and this effect was strongest in married parents, indicating that issues in the marriage are most likely to be the cause of increased loneliness rather than the arrival of a child.^23^ However, in contrast, a further study involving data from 17 nations found lower loneliness was associated with marital status.^24^ This study found that loneliness related to parenting status in men, but not in women; being married and having children was protective of male loneliness but not female loneliness. But in most nations, however, having children had no impact on adult loneliness, indicating that there may be cultural differences in the prevalence of parental loneliness.

Another three studies examined conceptual aspects of loneliness and whether the experience differs in motherhood. These studies used a methodology whereby participants were given a loneliness questionnaire (designed by the authors) and differences in responses across sub-scales were examined between mothers and women who were not parents. One study by Rokach^25^ found that pregnant women and new mothers had lower levels of emotional distress, social inadequacy and alienation, interpersonal isolation, and self-alienation in relation to loneliness when compared to women in the general population. Another study by Rokach^26^ found that pregnant women and new mothers were less likely to report experiencing loneliness that they felt was a result of their own personal inadequacies, such as mistrust or low self-esteem or social marginalisation (i.e. isolation and alienation) than women who were not parents. A further study, also by Rokach,^27^ examining coping with loneliness found that women who were not parents scored higher on reflection and acceptance, distancing and denial of loneliness than new parents and pregnant women. These studies indicate that causes of loneliness and strategies for coping may be different in parents than in other cohorts.

#### *Parents at increased risk of loneliness (n* *=* *80)*

Most of the included studies examined loneliness in specific cohorts of parents, demonstrating that some parents may be at an increased risk of experiencing loneliness. However, few of these studies had comparison or control groups, which makes it difficult to draw conclusions about whether these parents have higher loneliness or are at increased risk of loneliness.

The largest number of studies in this category related to loneliness in parents with a child with a chronic health condition or disability (*n* = 25). Many of these studies (*n* = 10) used a qualitative design, and loneliness in this group of parents was experienced due to a sense of helplessness, lack of psychosocial resources, feeling burdened by the child’s needs, lack of support from others or support available not meeting their needs, and changes in relationships with their partner.^28–31^ There were only three studies that compared loneliness in parents with a child with a chronic illness or disability to a control group that did not have a child with an illness or disability. In two of three studies, loneliness was higher in the parents with a child with a chronic illness or disability than the control group,^32,33^ but in one, there was no difference between the groups.^34^ A further six studies used frequency counting or content analysis and the percentage of parents with children with chronic illness or disability reporting loneliness ranged from 19.1% to 70%.^35–40^

Another group of parents identified as experiencing loneliness were immigrant or ethnic minority parents (*n* = 11). All of these studies involved mothers only, there were no comparison studies, and most used a qualitative design. Loneliness was experienced in these mothers due to an absence of support from their mother or mother-in-law. These mothers expressed a sense that the culture in the country they were in was different to their home country in the availability of support from kin and community in caring for their baby, which made them feel isolated, particularly in the postpartum period.^41–44^ Loneliness was particularly intensified when there were problems with their baby.^41^ Discrimination and language barriers further isolated them.^45,46^

There were several studies (*n* = 11) that examined loneliness in adolescent mothers, but evidence was less homogeneous and revealed conflicting findings. Two comparison studies found loneliness was higher in adolescent mothers than mothers in other age groups,^47,48^ but another found loneliness to be higher in non-parent adolescents than adolescents who were parents.^49^ In another study, adolescent mothers were no more likely to be lonely than mothers of other ages.^50^ Qualitative studies revealed that loneliness in adolescent mothers was linked to losing friendships; adolescents’ mothers did not experience loneliness if they were able to maintain existing friendships or make new ones.^51,52^

Single parents (*n* = 8) were also identified as experiencing loneliness, with studies showing between 8% and 21% of single parents reporting feeling lonely.^53–55^ Loneliness was experienced by single parents because of the absence of a partner and a lack of companionship (particularly someone to share experiences with).^56^ For some, the transition to single parenthood brought loneliness, but for others, it brought a sense of selfhood, freedom and liberation.^57^

There were some studies (*n* = 7) examining loneliness in first-time parents. Loneliness in this population was linked to finding parenthood unexpectedly difficult, feeling vulnerable as a parent, having fewer social interactions after becoming a parent and when first-time parents felt that the support received from their partner was superficial and/or that parenting responsibility rested with them.^58^

There were some studies that examined loneliness in low-income parents (*n* = 4) and mothers with poor health (*n* = 3) but were not sufficient in number to synthesise. Further studies explored loneliness in parents in relation to housing (e.g. living in a flat or sheltered accommodation; *n* = 2), partner violence/abuse (*n* = 2), returning to work after parental leave (*n* = 2), substance abuse (*n* = 2), being a gender variant parent (*n* = 2) or military wife (*n* = 1).

#### *Impacts of loneliness on health and wellbeing (n* *=* *33)*

##### Impacts on parent health and wellbeing (n = 14).

Studies that have examined the impacts of loneliness on parent health and wellbeing have only measured stress/distress and depression outcomes. Five studies examined relationships between parenting stress/distress and loneliness. Two of these studies used a correlational design and show cross-sectional associations between loneliness and parenting stress and distress.^59,60^ In a further cross-sectional study, mothers of different age children were surveyed and loneliness was found to be highest in preschool and middle school years and although the study did not examine an association with stress directly, stress followed a similar pattern of change across time as loneliness.^61^ In another qualitative study, parents who were experiencing burnout were recruited to explore their lived accounts of loneliness.^62^ That study found loneliness was associated with burnout through a sense of feeling strange and disconnected due to feelings of exhaustion. A further study^63^ examined the reasons for referral to parenting support services (i.e. demonstrating parental distress) and found that loneliness and low emotional wellbeing were the most common reasons for referral (38%). Findings here are limited because all the studies are cross-sectional so the direction of effect is not clear, it could be that parenting stress leads to loneliness or feeling lonely as a parent increases a parent’s stress/distress.

A further nine studies examined relationships between loneliness and depression in parents. Two qualitative studies with parents with postnatal depression found loneliness to be reported,^64,65^ with loneliness being due to discomfort with others and not feeling understood.^65^ In two cross-sectional studies comparing groups of mothers with depression symptoms or postnatal depression with those who were not depressed, we found that loneliness was more frequent or higher in mothers with depression.^66,67^ In one longitudinal study, loneliness predicted postnatal depression^68^ and in another loneliness was predictive of chronic depression in mothers.^69^ In a further longitudinal study, depression was higher in both mothers and fathers experiencing prolonged loneliness.^70^ However, in another study that included both mothers and fathers, loneliness was associated with depression, but marital dissatisfaction was a stronger predictor of depression than loneliness in mothers.^71^ A further study with fathers of children whose mothers have postnatal depression found that fathers developed loneliness as a result of a sense of not knowing whether their supportive efforts were working.^18^

##### Impacts on child’s health and wellbeing (n = 9).

Studies examining the impact of parental loneliness on child’s health and wellbeing are displayed in Table 2. Five of those studies used a cross-sectional design (i.e. measuring psychosocial variables in parent and child at the same time point), and the rest (*n* = 4) used a longitudinal design (typically measuring parent’s loneliness at one time point and child’s at another time point or series of timepoints). All nine studies used a loneliness measure, but these varied greatly. In four studies, impacts of fathers and mothers’ loneliness on their offspring were examined, but in five, only the impact of the mothers’ loneliness was examined. Findings across the studies show that loneliness in parents impacts child’s outcomes, but there are gender-specific effects. Mothers’ loneliness was associated with her child’s poorer problem-solving skills,^72^ internalising problems,^73,86^ social competence, hostility and fear of negative evaluation^79^ and social anxiety (but in girls only).^78^ Mothers and fathers’ loneliness impacted on peer-evaluated cooperating skills in girls.^75^ Mothers’ loneliness was associated with child’s loneliness, but not fathers’ loneliness in one cross-sectional study,^74^ whereas in another study, father’s loneliness was predictive of son’s persisting loneliness and mother’s loneliness was predictive of daughters.^77^ Only one study examined potential mediators of the relationship between parent’s and child’s loneliness finding an association between high parental loneliness and low parenting self-efficacy which was associated with children’s loneliness.^76^

#### *Loneliness and breastfeeding (n* *=* *2)*

There were two studies involving interviews with mothers which demonstrated that loneliness influences a women’s decision to stop breastfeeding. One qualitative study found that postpartum loneliness and sadness were due to mothers feeling that no one understood their difficulties with breastfeeding and that they had no one to support them.^87^ The other study used a lifeworld hermeneutical approach and found that women sought social connections as a means to mitigate loneliness aligned with their needs to either continue or stop breastfeeding.^88^ For women who wanted to or who had stopped breastfeeding, loneliness led to social withdrawal because of a fear of being detected as underperforming, useless and different; these women sought out others who had stopped breastfeeding to reinforce their choice. For others, to escape loneliness, they sought out others who could provide support to continue breastfeeding and their loneliness reduced as a result of these social connections and a sense of belonging.

#### *Child abuse/neglect (n* *=* *8)*

There were also some studies that examined relationships between loneliness and child abuse/neglect but these were quite dated, with publication dates ranging from 1980 to 2011 and all but one study conducted more than 10 years ago. In addition, all studies in this category were conducted in America thus lacking a cross-cultural comparison. All but one study examined loneliness in mothers who were in families identified as neglectful or at risk of child abuse, with the others examining mothers and fathers where parents are identified as abusers. All the studies in this category used a quantitative design and measured loneliness using a loneliness scale. Five used versions of the UCLA scale,^21^ two used the Loneliness subscale of the Child Abuse Potential Inventory (CAPI)^89^ and one used Emotional Social Loneliness and Isolation Scale.^90^

The relationship between loneliness and child abuse/neglect has been examined in these studies in two ways: (1) whether there is an association between loneliness and child abuse/neglect and/or whether loneliness predicts child abuse/neglect (*n* = 3) and (2) whether mothers in families identified as neglectful have higher loneliness (*n* = 5). The studies in this category were all cross-sectional, so although they do use regression models to look at predictors of abuse/neglect, the studies can only show an influence/association. In two out of the three association studies, loneliness was not associated with parental use of punishment^91^ and did not predict child neglect.^92^ Whereas in the other study, loneliness predicted child abuse potential in mothers with disabled children.^93^ Where level of loneliness was compared to a control group, loneliness was higher in neglectful parents,^94^ abusing parents^95^ and mothers in families identified as neglectful.^96,97^ In families that were identified as at risk of child abuse, loneliness was higher in mothers where fathers were not involved than mothers with a resident father.^98^

#### *Intervention studies (n* *=* *14)*

The review identified 14 intervention studies with parents that measured loneliness as an outcome (see Table 3). Most of these intervention studies were conducted with new parents, with some specifically conducted with mothers who had postnatal depression or who were at risk of child abuse/neglect. None of the interventions were specifically designed to reduce loneliness, but one was designed to target social isolation in parents with children with cerebral palsy^100^ and another to increase social support in parents at risk of child maltreatment.^99^ Most studies used a quantitative design, with one study using a mixed design and another a qualitative design. All but one intervention study measured loneliness using UCLA,^21^ but the version used varied across the studies. Only three of the studies were noted as randomised trials.^101–103^ In relation to effectiveness, only 6 of the 14 intervention studies showed reductions in loneliness. Interventions that reduced or showed promise of reducing loneliness involved home visiting peer support, tele-health involving e-meeting forum with HCP and peers, universally provided child development parenting programme, interpersonal skills training and short-term cognitive therapy.

**Table 3:** Intervention studies measuring loneliness as an outcome

Author	Year	Sample	Intervention	Country	Data collection waves	Findings
*Studies showing reductions in loneliness*						
Chan	2005	New mothers (locality with high incidence of child abuse)	Home visiting peer support	China	Before receiving service and 1 year later	Loneliness reduced in the intervention group but not in the control group
Nystrom	2006	New mot hers	Telehealth, involving e-meeting forum with Health Care Professional	Sweden		Mothers reported having good social networks but spent most of the day alone with their children; meeting others in a similar situation made them feel less alone and friends were made in the group
Richey et al.^99^	1991	Mothers at risk for child maltreatment	Interpersonal skills training	US	Pre- and post-training sessions	Slight decrease in loneliness was reported pre- and post-training (no statistical analysis conducted – only 6 mothers)
Skar	2015	New mothers	Child development parenting programme	Norway	Immediately after, 6–12 months after	Greater reduction in loneliness in the intervention group than the control group
Sorenson	2003	New mothers (traumatic childbirth provider interactions)	Short-term cognitive group therapy	US	Pre- and postintervention	Loneliness was reduced pre- to postintervention
Zare et al.^100^	2017	Mothers with children with CP	Self-management empowerment intervention	Iran	Pre- and postintervention	Intervention shows promise of reducing loneliness (independent *t*-test used rather than ANOVA so difficult to be conclusive)
*Studies not showing reductions in loneliness*						
Dennis et al.^101^	2009	New mothers (high postnatal depression)	Telephone peer support	Canada	Baseline, 12 weeks and 24 weeks	No difference in loneliness between intervention group and control group
Dennis^102^	2003	New mothers (high depression)	Peer support by lay volunteers	Canada	Baseline and 8 weeks later	No difference in loneliness between the control and intervention group
Hudson	2012	New mothers	Online discussion forum with Health Care Professional	US	1 week, 6 weeks, 3 months and 6 months following birth	No differences across the intervention period in loneliness or differences between the intervention and control group
Razani et al.^103^	2018	Low-income parents	Park prescription	US	Baseline, 1 month and 3 months later	Reduction in loneliness in both groups from baseline and 3 months later, but no differences between the groups
Shorey	2019	New mothers at risk of postnatal depression	Technology-based peer-support	Singapore	1 month and 3 months postpartum	No differences in loneliness scores and no difference in change in loneliness scores
Tuominen	2016	New mothers	Relational continuity of care	Finland		Relational continuity of care associated with higher levels of mothers’ emotional loneliness
White	1987	Single parents	Peer support group	Australia		The old peer support and never had peer support groups were very similar on loneliness and new group reported higher levels of loneliness
Yarnoz	2008	Divorced parents	Attachment-based intervention	Spain	Pre- and postintervention	No differences in loneliness pre- and postintervention

## Discussion

The aim of this scoping review was to map existing literature to establish what is already known about parental loneliness. Although there is a scarcity of studies that have specifically focussed on understanding loneliness in parenthood, there are a large number of studies that have included loneliness as an outcome or have examined the lived experience of parents in specific populations (e.g. adolescent parents) where loneliness has been identified.

Studies show that loneliness during parenthood is stable and may be different to loneliness experienced in other cohorts.^22,25–27^ However, there was a lack of conceptual studies to identify the key underlying mechanisms associated with parental loneliness, and no prospective studies that commenced in the preconception period to help understand whether and how loneliness changes over parenthood. It is plausible to assume that while parenthood may help to mitigate loneliness as there is a dependent infant to care for, there is evidence to suggest that loneliness may be exacerbated by becoming a parent. Other transitory phases in life, where changes are made in social connections and friendships, are also associated with increased loneliness, such as the transition to university^104^ or retirement.^105^

Wider research indicates, and is reflected in some of the included studies in the scoping review,^61,63,86^ that loneliness is associated with increased risks of depression, anxiety and increased stress.^3,106^ Our findings also support those from other cohorts in terms of reciprocal relationships between loneliness and depression,^107^ with loneliness in parents found to be predictive of depression^86^ and depression predictive of loneliness.^70^ However, the direction of this effect has not yet been examined in this population, and further research (i.e. using cross-lagged designs where reciprocal relationships between loneliness and depression over time can be examined enabling direction of effect to be explored) is needed. While loneliness has been associated with poor physical health in other cohorts,^4^ we found no studies that examined the association between loneliness in parents and physical health outcomes; thereby identifying a further gap where more research is needed.

Parental loneliness, similar to other evidence of the negative impacts of poor parental mental health,^108^ was associated with adverse repercussions on child’s health and wellbeing, in relation to breastfeeding cessation, mental health and social competence. The findings from the scoping review also indicate the potential for some gender-specific effects of intergenerational transmission of loneliness and social competence from parent to child. This is similar to other research where gender-specific effects have been found for the intergenerational transmission of internalising behaviours (depression and irritability)^109^ and depression,^110^ but because there are few studies, this warrants further investigation.

The findings that parental loneliness was also associated with child abuse and neglect need to be treated with caution as the evidence base only includes cross-sectional studies and other factors had not been accounted for (e.g. social isolation, being in an abusive relationship or poor mental health). Furthermore, while it is perhaps not surprising that parents who face additional challenges (e.g. who have children with chronic illness or disability, immigrant or ethnic minority parent, single parents) are at increased risk of loneliness, the evidence is not conclusive due to a lack of comparison studies and further research is needed. It may also prove beneficial to consider factors that can help mitigate adversities, rather than assumptions that all outcomes associated with loneliness will be negative, and to identify more resilience-based factors that can help to combat loneliness, such as personal or community assets.^111,112^ Further research is also needed with fathers and wider partners to assess differences between the parents, and international studies to explore cross-national and cross-cultural differences.

While interventions included in this scoping review have not generally been designed to reduce loneliness, this work has identified some key mechanisms of effectiveness to consider within future intervention designs. These include developing communication skills and forming social connections via engaging women in peer support. This aligns with wider literature that reveals that peer support provides feelings of validation, normalisation and reassurance,^113^ and helps to reduce negative emotional impacts (such as social isolation) through building social connections and networks.^114^

While it will be important to conduct further systematic reviews and meta-syntheses in this area, particularly in relation to conceptual aspects and potential mechanisms of parental loneliness, this scope of the literature highlights some potential common factors of experiencing loneliness in parenthood. The evidence appears to point to parents being at increased risk of loneliness if they have few or no peers in a similar situation with whom they can share their particular circumstances, have negative thoughts towards themselves, or have reduced social support or ability to seek extra support. These findings overlap with those in the wider literature with other cohorts where loneliness has been associated with a lack of belonging, internalising attributional style, low self-worth and lacking emotional support.^115,116^ Although, there is also evidence that there may be some differences in the causes and experiences of loneliness in parents^25–27^ that warrant further investigation to ensure we have a nuanced understanding of those who are at risk of experiencing loneliness and how they experience loneliness overtime, and to help inform appropriate and relevant interventions.

### Strengths and limitations of the review

The strengths of this review are its broad and comprehensive approach that meant that a wide range of relevant studies were included. We also only focused on studies that measured loneliness rather than include other related social connection measures such as social network size and social support. Further reviews could examine specific aspects of parental loneliness and social connection more generally to help understand the underpinning mechanisms that explain loneliness in parenthood and to inform future interventions. The end date of the review period was restricted to February 2020, to prevent COVID-related studies being included. While loneliness is undoubtedly a key feature of the current pandemic, our aim was to elicit insights into parental loneliness per se, rather than loneliness created via enforced isolation and restricted social connections. As this is a scoping review, we did not assess for quality, which means that studies of low quality may have been included. As we intended to map and synthesis extant literature on a wide topic area using disparate methods, a meta-analysis was not deemed appropriate, which means that the review involves a narrative synthesis of the findings focussed on general themes and patterns in the data. The review does however provide the first, comprehensive understanding of the work undertaken in this area and offers insights to direct future research, highlighting gaps in the existing literature.

## Supplemental Material

sj-docx-1-rsh-10.1177_17579139211018243 – Supplemental material for Experiencing loneliness in parenthood: a scoping review Click here for additional data file. Supplemental material, sj-docx-1-rsh-10.1177_17579139211018243 for Experiencing loneliness in parenthood: a scoping review by R Nowland, G Thomson, L McNally, T Smith and K Whittaker in Perspectives in Public Health
