# Supplementary material for: Experiencing loneliness in parenthood: a scoping review
Source: Perspect Public Health. 2021 Jul 21;141(4):214–25. doi: 10.1177/17579139211018243 (PMC8580382; doi:10.1177/17579139211018243)
Supplement: sj-docx-1-rsh-10.1177_17579139211018243 – Supplemental material for Experiencing loneliness in parenthood: a scoping review [file sj-docx-1-rsh-10.1177_17579139211018243.docx]

**Supplementary information 1**

Search Strategy & Results:

EMBASE

1. ((mother* or maternal or parent* or paternal or father*) and (lonel* or "perceived social isolat*")).mp. [mp=title, abstract, heading word, drug trade name, original title, device manufacturer, drug manufacturer, device trade name, keyword, floating subheading word, candidate term word] = 1151

2. limit 1 to (human and english language) = **970**

Medline

1. ((mother* or maternal or parent* or paternal or father*) and (lonel* or "perceived social isolat*")).mp. [mp=title, abstract, heading word, drug trade name, original title, device manufacturer, drug manufacturer, device trade name, keyword, floating subheading word, candidate term word] Limiters: human and english language = **621**

PSYCHINFO

AB ( mother* OR maternal OR parent* OR paternal OR father* ) AND AB lonel* limiters: English; Population Group: Human, peer reviewed, English language, exclude dissertations = **1140**

Note: the search would not run with “perceived social isolat*” so this was removed – PSYCHOINFO won’t run if any of the searches result in zero.

CINHAL

( mother* or maternal or parent* or paternal or father* ) AND ( lonel* or "perceived social isolat*" ) Limiters – English language; Peer Reviewed; Exclude MEDLINE records; Human = **210**

Web of Science

(TS=(mother* or maternal or parent* or paternal or father*) AND TS=(lonel*)) *AND* **LANGUAGE:** (English) *AND* **DOCUMENT TYPES:** (Article)

*Indexes=SCI-EXPANDED, SSCI, A&HCI, CPCI-S, CPCI-SSH, ESCI Timespan=All years* **= 965**

SCOPUS

TITLE-ABS-KEY (mother* OR maternal OR parent* or *paternal* OR father* AND lonel*) = **165**

**Supplementary Information 2**

Table 2. Description of included articles

| First Author | Year | Country | Parents | Design | Main focus |
| --- | --- | --- | --- | --- | --- |
| Aching^118^ | 2016 | Brazil | mothers | qual | Parenthood experiences of mothers living in a shelter |
| Alaee^28^ | 2015 | Iran | both | qual | Experiences of parenting a child with cerebral palsy |
| Alvik^73^ | 2014 | Norway | mothers | quant | Predictors of low developmental scores in child |
| Al-Yagon^74^ | 2007 | Israel | mothers | quant | Predictors of child's socio-emotional and behavioural adjustment |
| Atkins^53^ | 2018 | USA | mothers | qual | Expressions and feelings associated with depression in black single mothers |
| Au^59^ | 2008 | China | both | quant | Factors influencing parenting stress, parenting distress and parent-child dsyfunction |
| Ayers^119^ | 2019 | UK | mothers | qual | Key stressors during the perinatal period |
| Badaru^66^ | 2013 | Nigeria | mothers | quant | Factors associated with mothers depression |
| Barth^49^ | 1983 | USA | mothers | quant | Psychological impacts of adolescent pregnancy and motherhood |
| Beck^644^ | 1992 | USA | mothers | qual | Experiences of mothers with postpartum depression |
| Berry^60^ | 1995 | USA | both | quant | Scale measurement (parenting stress) |
| Björk^120^ | 2005 | Sweden | both | qual | Experiences of families with a child with diagnosis of cancer |
| Bloom^121^ | 2012 | USA | mothers | qual | Stresses for low income mothers |
| Boman^34^ | 2003 | Sweden | both | quant | Psychological predictors of disease-related distress in parents of children with a cancer diagnosis |
| Bonman^122^ | 2004 | Sweden | both | quant | Distress in parents with children with cancer or diabetes |
| Boxill^123^ | 1987 | USA | mothers | qual | Experiences of black adolescents |
| Burnes^30^ | 2008 | USA | mothers | qual | Experiences of African and African Caribbean mothers with children with sickle cell disease |
| Cameron^124^ | 2002 | Canada | mothers | qual | Motivations for parents on welfare state to join parent mutual aid organisations |
| Cannon-Bonventre^17^ | 1979 | USA | both | qual | Experiences of adolescent parents |
| Chan^125^ | 2005 | China | mothers | quant | Evaluation of home visiting programme to prevent child abuse |
| Charter^19^ | 2018 | Australia | fathers | qual | Experiences of parenthood for transgender men |
| Chesser^47^ | 1981 | USA | mothers | quant | Loneliness in adolescent mothers |
| Childs^35^ | Fal | USA | mothers | quant | Feelings of mothers after the birth of a retarded child |
| Cronin^126^ | 2002 | Ireland | mothers | qual | Experiences of first-time mothers |
| Cutrona^127^ | 1986 | USA | mothers | quant | Social network size as a predictor of loneliness and social provisions in new mothers |
| Dennis^96^ | 2009 | Canada | mothers | quant | Evaluation of peer support intervention for mothers with high postnatal depression scores |
| Dennis^97^ | 2003 | Canada | mothers | quant | Effectiveness of a peer support intervention to reduce depression in new mothers |
| Dykstra^128^ | 2009 | Netherlands | fathers | quant | Well-being influences of parenthood in men |
| Ekas^129^ | 2016 | USA | mothers | quant | Influences on depression in mothers with autistic children |
| Ellis^20^ | 2015 | USA |  | qual | Experience of gender variant gestation parents |
| Ellis-Sloan^51^ | 2019 | UK | mothers | qual | Experience of teenage mothers |
| Eni^130^ | 2014 | Canada | mothers | qual | Determinants of breastfeeding in First Nations women |
| Ergun^131^ | 2012 | Turkey | mothers | quant | Difficulties experienced by mothers who have children with intellectual disabilities |
| Essex^132^ | 2007 | USA | mothers | quant | Predictors of loneliness in substance abusing, court-involved mothers |
| Fabian^133^ | 2006 | Sweden | mothers | quant | Predictors of non-attending parental education classes for first time mothers |
| Farzizadeh^134^ | 2018 | Iran | mothers | qual | Impacts of living with a drug addicted father |
| Florian^32^ | 1991 | Israel | mothers | quant | Loneliness and social support in mothers with chronically ill children |
| Forinder^135^ | 2010 | Sweden | mothers | qual | Experiences of parents with children surviving a brain tumour |
| Gaudin^89^ | 1993 | USA | mothers | quant | Loneliness, depression and stress in neglectful families |
| Gladow^136^ | 1986 | USA | mothers | quant | Impact of informal support systems in low income single parents |
| Gosztyla^137^ | 2017 | Poland | both | quant | Determinants of loneliness in parents of children with autism |
| Gosztyła^138^ | 2019 | Poland | both | quant | Role of actual and perceived social support in the relationship between extraversion and loneliness in parents of children with ASD and with ID |
| Gove^139^ | 1977 | USA | both | quant | Effects of employment, number of children and age of youngest child on the feeling lonely |
| Graffigna^140^ | 2013 | Italy | both | qual | Experience of families with children with tuberous sclerosis complex |
| Grootenhuis^141^ | 1997 | Netherlands | both | quant | Influence of control strategies used by parents of children with cancer on emotional adjustment |
| Gulhati^142^ | 1998 | UK | mothers | quant | Emotions of mothers with children with pain symptoms without obvious pathology |
| Halford^143^ | 2013 | Australia | both | quant | Influences on psychological distress after partner separation |
| Halsa^144^ | 2018 | Norway | mothers | qual | Experiences of mothers with mental health conditions |
| Hamama-Raz^145^ | 2015 | Israel | both | quant | Factors influencing quality of life in parents with children with epilepsy |
| Hattar-Pollara^45^ | 1995 | USA | mothers | qual | Experiences of immigrant mothers |
| Henwood^79^ | 1994 | USA | both | quant | Intergenerational transmission of loneliness |
| Hoban^41^ | 2013 | Australia | mothers | qual | Experiences of immigrant mothers |
| Hubert^62^ | 2018 | Belgium | mothers | qual | Experiences of mothers with parental burnout |
| Hudson^146^ | 2012 | USA | mothers | quant | Effectiveness of social media intervention new mothers |
| Hudson^147^ | 2000 | USA | mothers | quant | Depression, self-esteem, loneliness and social support in adolescent mothers |
| Hudson^148^ | 2016 | USA | mothers | quant | Social support, self-esteem and loneliness in low income African American single mothers |
| Igarashi^149^ | 2013 | Japan | mothers | quant | Immigrants' experiences of maternity care |
| Junttila^22^ | 2013 | Finland | both | quant | Stability of parents' social and emotional loneliness in pregnancy, infanthood, to toddler years |
| Junttila^81^ | 2007 | Finland | both | quant | Parents loneliness and parenting self-efficacy and child's social and academic behaviour |
| Junttila^78^ | 2009 | Finland | both | quant | Impact of parents' loneliness on child's cooperation skills and social and emotional loneliness |
| Junttila^70^ | 2015 | Finland | both | quant | Parents’ social and emotional loneliness during pregnancy, infancy, and early childhood |
| Katz^54^ | 1991 | Israel | mothers | quant | Well-being of divorced and widowed mothers since husband's death or divorce |
| Keizer^23^ | 2010 | Netherlands | both | quant | Impact of partner status and work hours on well-being of parents who are transitioning into parenthood |
| Khan^150^ | 2018 | Canada | mothers | quant | Psychosocial experiences and needs of women with HIV during pregnancy and early postpartum. |
| Kim^151^ | 2011 | Korea | mothers | qual | Survival experiences of mothers of children who had reported their partner as having abused them |
| Kirkham^88^ | 1986 | USA | mothers | quant | Factors influencing child abuse potential in mothers with disabled children |
| Klajmon-Lech^152^ | 2016 | Poland | both | qual | Parents experiences of early support and intervention when child has a diagnosis of a rare genetic condition |
| Kruse^74^ | 2014 | USA | mothers | quant | Predictors of postpartum depression |
| Kulkarni^153^ | 2009 | USA | mothers | qual | Influence of interpartner violence on relationships in adolescent mothers |
| Layton^154^ | 2007 | UK | mothers | qual | Experiences of immigrant mothers |
| LeDrew^155^ | 2018 | Canada | mothers | qual | Experiences of new mothers whose partner works away from home |
| Lee^58^ | 2017 | UK | mothers | qual | First time mothers’ experiences of loneliness and expected and actual experiences motherhood |
| Leyser^37^ | 1988 | Isarel | both | qual | Experiences of families with a disabled child living in a kibbutz |
| Luoma^68^ | 2019 | Finland | mothers | quant | Loneliness in first-time mothers |
| Luoma^69^ | 2015 | Finland | mothers | quant | Antenatal factors associated with chronic maternal depressive symptoms |
| Luthar^61^ | 2016 | USA | mothers | quant | Challenges for mothers in the infancy and middle school years |
| Lutz^71^ | 2002 | USA | both | quant | Factors influencing depression in first time parents |
| Mandai^48^ | 2018 | Japan | mothers | quant | Predictors of loneliness among mothers raising children |
| Manor-Binyamini^31^ | 2018 | Israel | mothers | qual | Immigrant mothers’ experiences of parenting a children with autism |
| Maurice-Stam^156^ | 2008 | Netherlands | both | quant | Parents adjustment and coping following child's treatment for cancer |
| Mayers^157^ | 2005 | USA | mothers | qual | Existential issues and coping in low-income mothers with HIV |
| Medora^86^ | 2001 | USA | mothers | quant | Attitudes of low-income mothers toward parenting, the potential for child abuse, and parental satisfaction |
| Milner^90^ | 1980 | USA | both | quant | Differences in abusing and non-abusing parents |
| Nahas^158^ | 1999 | Australia | mothers | qual | Meanings and expressions of postpartum depression among immigrant mothers |
| Nahas^42^ | 1999 | Australia | mothers | qual | Experience of postpartum depression in immigrant mothers |
| Nystrom^159^ | 2006 | Sweden | mothers | qual | New mothers’ experiences of an intervention using telehealth for parenting support |
| Oakley^63^ | 1998 | UK | mothers | quant | Characteristics of mothers referred to Home Start and Newpin and referral reasons |
| Omer-Salim^160^ | 2014 | India | mothers | qual | Manifestations of agency in combining breastfeeding and employment amongst health workers |
| Ornelas^46^ | 2009 | USA | mothers | qual | Factors influencing depressive symptoms among immigrant mothers |
| Palmér^83^ | 2015 | Sweden | mothers | qual | Women’s decisions to continue or cease breastfeeding |
| Paris^161^ | 2005 | USA | mothers | qual | Experiences of new mothers and their perceptions of the home-visitor relationship |
| Pelentsov^38^ | 2016 | Australia, New Zealand | both | quant | Supportive care needs of parents caring for a child with a rare disease |
| Polansky^91^ | 1985 | USA | mothers | quant | Loneliness in parents who are neglectful |
| Polansky^92^ | 1985 | USA | mothers | quant | Loneliness in parents who are neglectful |
| Razani^98^ | 2018 | USA | both | quant | Evaluation of park prescriptions for parents at a low-income clinic |
| Richey^95^ | 1991 | USA | mothers | quant | Feasilbity of using Interpersonal Skill Training in mothers at risk of child maltreatment |
| Richman^162^ | 1974 | UK | mothers | quant | Effect of living in high-rise flats, compared to living in other types of housing |
| Ritchie^55^ | 1980 | New Zealand | mothers | quant | Social characteristics of a group of single mothers |
| Rokach^25^ | 2004 | Canada | mothers | quant | Qualitative aspects of loneliness in pregnant women and new mothers |
| Rokach^26^ | 2007 | Canada | mothers | quant | Perceived causes of loneliness in pregnant women and new mothers |
| Rokach^27^ | 2005 | Canada | mothers | quant | Differences in loneliness coping strategies in pregnant women and new mothers |
| Rolls^163^ | 2001 | Australia | both | qual | Experiences of mothers having a child with a sleep problem |
| Rozenzwieg^164^ | 1981 | Israel | mothers | qual | Experiences of military wives attending a Family and Child Centre |
| Russo^43^ | 2015 | Australia | mothers | qual | Experiences of immigrant mothers |
| Salo^80^ | 2020 | Finland | both | quant | Intergenerational transmission of loneliness |
| Schlesinger^56^ | 1991 | Canada | mothers | qual | Experiences of immigrant mothers |
| Shahar^87^ | 2001 | USA | mothers | quant | Personality, loneliness and depression as Predictors of Child Neglect |
| Shapiro^93^ | 2011 | USA | mothers | quant | Fathers and father-related family dynamics in families identified at risk for child abuse |
| Shorey^165^ | 2019 | Singapore | mothers | quant | Effectiveness of a technology-based peer-support program in new mothers at risk of developing PND |
| Silverstein^166^ | 2010 | USA | mothers | qual | Low-income urban mothers’ explanations of feelings of sadness, stress or demoralization |
| Skar^167^ | 2015 | Norway | both | quant | Impact of International Child Development Programme |
| Sorenson^168^ | 2003 | USA | mothers | quant | Effectiveness of cognitive group therapy in mothers who experienced childbirth trauma |
| Stack^24^ | 1998 | various | both | quant | Influence of marital and parenting status on loneliness |
| Stam^33^ | 2006 | Netherlands | both | quant | Well-being of parents following child's successful cancer treatment |
| Stednitz^77^ | 2006 | USA | mothers | quant | Influence of mothers' social functioning on child's social anxiety |
| Stewart^169^ | 2015 | Canada | both | mixed | Challenges faced by refugee new parents |
| Stewart^170^ | 2015 | Canada | both | mixed | Evaluation of a social support intervention for new parents who are refugee |
| Tcheng-Laroche^57^ | 1979 | Canada | mothers | qual | Lifestyle, health and stress in middle income divorced mothers |
| Tirgari^171^ | 2019 | Iran | mothers | quant | Experiences of Iranian Teen Mothers with parenting Stress |
| Tuominen^172^ | 2016 | Finland | both | quant | Effects of continuity of care on parents' parenting self-efficacy |
| Ugarriza^65^ | 2002 | USA | mothers | qual | Postpartum depressed women’s explanations for their depression |
| Valdez^72^ | 2019 | USA | fathers | qual | Fathers’ experiences with maternal depression in an immigrant sample |
| Van Dongenmelman^39^ | 1995 | Netherlands | both | quant | Late psychosocial consequences for parents of children who survived cancer |
| Van Oers^40^ | 2019 | Netherlands | both | quant | Well-being of parents of children on home parenteral nutrition |
| Vicary^50^ | 2001 | USA | mothers | quant | Psychosocial functioning of adolescent mothers transitioning to parenthood |
| Volk^44^ | 2009 | USA | mothers | qual | Experiences of immigrant mothers |
| White^173^ | 1987 | Australia | both | mixed | Evaluation of parents without partners programme |
| Yako^52^ | 2007 | South Africa | mothers | mixed | Adolescent mothers’ reasons for becoming pregnant and experiences of pregnancy and parenting |
| Yarnoz^174^ | 2008 | Spain | both | quant | Effectiveness of an attachment-based intervention to reduce loneliness, fear and grief in divorced parents |
| Zafar^76^ | 2015 | India | mothers | quant | Differences in psychosocial factors in divorced and married mothers |
| Zaidi^67^ | 2017 | India | mothers | quant | Risk factors for postpartum depression |
| Zare^94^ | 2017 | Iran | mothers | quant | Intervention for aggression and loneliness in mothers with children with CP |
| Zekeri^175^ | 2007 | USA | mothers | quant | Prevalence of loneliness in low income, single parent mothers |

Full reference list for studies is available in supplementary materials

**Supplementary Information 3**

**Full Reference List**

1. Peplau LA. Perspective on loneliness. Loneliness: A Sourcebook of Current Theory, Research and Therapy. 1982.
2. Van Baarsen B, Snijders TA, Smit JH, Van Duijn MA. Lonely but not alone: Emotional isolation and social isolation as two distinct dimensions of loneliness in older people. Educational and Psychological Measurement. 2001 Feb;61(1):119-35.
3. Wang J, Mann F, Lloyd-Evans B, Ma R, Johnson S. Associations between loneliness and perceived social support and outcomes of mental health problems: a systematic review. BMC psychiatry. 2018 Dec 1;18(1):156.
4. Ong AD, Uchino BN, Wethington E. Loneliness and health in older adults: A mini-review and synthesis. Gerontology. 2016;62(4):443-9.
5. Gerst-Emerson K, Jayawardhana J. Loneliness as a public health issue: the impact of loneliness on health care utilization among older adults. American journal of public health. 2015 May;105(5):1013-9.
6. Action for Children. It starts with hello: A report into the impact of loneliness in children, young people and families. London; 2017
7. Peters MD, Godfrey CM, Khalil H, McInerney P, Parker D, Soares CB. Guidance for conducting systematic scoping reviews. International journal of evidence-based healthcare. 2015 Sep 1;13(3):141-6.
8. Arksey, H. and O'Malley, L., 2005. Scoping studies: towards a methodological framework. International journal of social research methodology, 8(1), pp.19-32.
9. Levac D, Colquhoun H, O'Brien KK. Scoping studies: advancing the methodology. Implementation science. 2010 Dec 1;5(1):69.
10. Ouzzani M, Hammady H, Fedorowicz Z, Elmagarmid A. Rayyan—a web and mobile app for systematic reviews. Systematic reviews. 2016 Dec 1;5(1):210.

Polanin, J. R., Pigott, T. D., Espelage, D. L., & Grotpeter, J. K. (2019). Best practice guidelines for Abstract screening large‐evidence systematic reviews and meta‐analyses. *Research Synthesis Methods*, *10*(3), 330-342.

Gartlehner G, Affengruber L, Titscher V, Noel-Storr A, Dooley G, Ballarini N, König F. Single-reviewer abstract screening missed 13 percent of relevant studies: a crowd-based, randomized controlled trial. Journal of clinical epidemiology. 2020 May 1;121:20-8.

Waffenschmidt S, Knelangen M, Sieben W, Bühn S, Pieper D. Single screening versus conventional double screening for study selection in systematic reviews: a methodological systematic review. BMC medical research methodology. 2019 Dec;19(1):1-9.

1. Daudt HM, van Mossel C, Scott SJ. Enhancing the scoping study methodology: a large, inter-professional team’s experience with Arksey and O’Malley’s framework. BMC medical research methodology. 2013 Dec 1;13(1):48.
2. Rokach A. Loneliness among loved ones: Alienation in the family. Psychology and Education: An Interdisciplinary Journal. 2003; 40(3-4): 1–18.
3. Sha'ked A, Rokach A, Morris R. Marital distress, loneliness and children's adjustment. Psychology and Education: An Interdisciplinary Journal. 2013; 50(3-4): 11–18.
4. Cannon-Bonventre K, Kahn J. Interviews with adolescent parents: looking at their needs. Children today. 1979;8(5):17.
5. Valdez CR, Martinez E. Mexican immigrant fathers’ recognition of and coping with maternal depression: The influence of meaning-making on marital and co-parenting roles among men participating in a family intervention. Journal of Latinx Psychology. 2019 Apr 15.
6. Charter R, Ussher JM, Perz J, Robinson K. The transgender parent: Experiences and constructions of pregnancy and parenthood for transgender men in Australia. International Journal of Transgenderism. 2018 Jan 2;19(1):64-77.
7. Ellis SA, Wojnar DM, Pettinato M. Conception, pregnancy, and birth experiences of male and gender variant gestational parents: it's how we could have a family. Journal of Midwifery & Women's Health. 2015 Jan;60(1):62-9.
8. Russell D, Peplau LA, Cutrona CE. The revised UCLA Loneliness Scale: concurrent and discriminant validity evidence. Journal of personality and social psychology. 1980 Sep;39(3):472.
9. Junttila N, Ahlqvist-Björkroth S, Aromaa M, Rautava P, Piha J, Vauras M, Räihä H. Mothers and Fathers Loneliness During Pregnancy, Infancy, and Toddlerhood. Psychol Educ J. 2013;50:98-104.
10. Keizer R, Dykstra PA, Poortman AR. The transition to parenthood and well-being: the impact of partner status and work hour transitions. Journal of family psychology. 2010 Aug;24(4):429.
11. Stack S. Marriage, family and loneliness: A cross-national study. Sociological perspectives. 1998 Jun;41(2):415-32.
12. Rokach A. Giving life: Loneliness, pregnancy and motherhood. Social Behavior and Personality: an international journal. 2004 Jan 1;32(7):691-702.
13. Rokach A. Self-perception of the antecedents of loneliness among new mothers and pregnant women. Psychological reports. 2007 Feb;100(1):231-43.
14. Rokach A. Coping with loneliness during pregnancy and motherhood. Psychology and Education: An Interdisciplinary Journal. 2005;42(1): 1–12.
15. Alaee N, Shahboulaghi FM, Khankeh H, Kermanshahi SM. Psychosocial challenges for parents of children with cerebral palsy: A qualitative study. Journal of Child and Family Studies. 2015 Jul 1;24(7):2147-54.
16. Björk M, Wiebe T, Hallström I. Striving to survive: Families’ lived experiences when a child is diagnosed with cancer. Journal of Pediatric Oncology Nursing. 2005 Sep;22(5):265-75.
17. Burnes DP, Antle BJ, Williams CC, Cook L. Mothers raising children with sickle cell disease at the intersection of race, gender, and illness stigma. Health & Social Work. 2008 Aug 1;33(3):211-20.
18. Manor-Binyamini I, Shoshana A. Listening to Bedouin mothers of children with autism. Culture, Medicine, and Psychiatry. 2018 Jun 1;42(2):401-18.
19. Florian V, Krulik T. Loneliness and social support of mothers of chronically ill children. Social Science & Medicine. 1991 Jan 1;32(11):1291-6.
20. Stam H, Grootenhuis MA, Brons PP, Caron HN, Last BF. Health‐related quality of life in children and emotional reactions of parents following completion of cancer treatment. Pediatric Blood & Cancer. 2006 Sep;47(3):312-9.
21. Boman K, Lindahl A, Björk O. Disease-related distress in parents of children with cancer at various stages after the time of diagnosis. Acta Oncologica. 2003 Mar 1;42(2):137-46.
22. Childs RE. Maternal psychological conflicts associated with the birth of a retarded child. Maternal-child nursing journal. 1985.
23. Ergün S, Ertem G. Difficulties of mothers living with mentally disabled children. J Pak Med Assoc. 2012
24. Leyser Y, Margalit M, Avraham Y. Families of disabled children in the Israeli kibbutz: A community which provides for all needs. The Exceptional Child. 1988 Nov 1;35(3):165-77.
25. Pelentsov LJ, Fielder AL, Laws TA, Esterman AJ. The supportive care needs of parents with a child with a rare disease: results of an online survey. BMC family practice. 2016 Dec 1;17(1):88.
26. Van Dongen-Melman JE, Pruyn JF, Groot AD, Koot HM, Hählen K, Verhulst FC. Late psychosocial consequences for parents of children who survived cancer. Journal of pediatric psychology. 1995 Oct 1;20(5):567-86.
27. van Oers H, Haverman L, Olieman J, Tabbers M, Grootenhuis MA. Health-related quality of life and distress of parents of children on Home Parenteral Nutrition. In Quality of Life Research. 2016 Oct 1;25: 196-196.
28. Hoban E, Liamputtong P. Cambodian migrant women's postpartum experiences in Victoria, Australia. Midwifery. 2013 Jul 1;29(7):772-8.
29. Nahas VL, Hillege S, Amasheh N. Postpartum depression: The lived experiences of Middle Eastern migrant women in Australia. Journal of nurse-midwifery. 1999 Jan 1;44(1):65-74.
30. Russo A, Lewis B, Joyce A, Crockett B, Luchters S. A qualitative exploration of the emotional wellbeing and support needs of new mothers from Afghanistan living in Melbourne, Australia. BMC pregnancy and childbirth. 2015 Dec;15(1):1-3.
31. Volk L. “Kull wahad la haalu” Feelings of Isolation and Distress among Yemeni Immigrant Women in San Francisco's Tenderloin. Medical Anthropology Quarterly. 2009 Dec;23(4):397-416.
32. Hattar-Pollara M, Meleis AI. The stress of immigration and the daily lived experiences of Jordanian immigrant women in the United States. Western Journal of Nursing Research. 1995 Oct;17(5):521-39.
33. Ornelas IJ, Perreira KM, Beeber L, Maxwell L. Challenges and strategies to maintaining emotional health: Qualitative perspectives of Mexican immigrant mothers. Journal of Family Issues. 2009 Nov;30(11):1556-75.
34. Chesser B, Woodward JC, Bauermeister M, Parkhurst AM. Loneliness Among Low‐Income, Single Adolescent Mothers. Home Economics Research Journal. 1981 Jun;9(4):374-81.
35. Mandai M, Kaso M, Takahashi Y, Nakayama T. Loneliness among mothers raising children under the age of 3 years and predictors with special reference to the use of SNS: a community-based cross-sectional study. BMC women's health. 2018 Dec 1;18(1):131.
36. Barth RP, Schinke SP, Maxwell JS. Psychological correlates of teenage motherhood. Journal of Youth and Adolescence. 1983 Dec 1;12(6):471-87.
37. Vicary JR, Corneal DA. A comparison of young women's psychosocial status based on age of their first childbirth. Family & community health. 2001 Jul 1;24(2):73-84.
38. Ellis-Sloan K, Tamplin A. Teenage mothers and social isolation: The role of friendship as protection against relational exclusion. Social Policy and Society. 2019 Apr 1;18(2):203-18.
39. Yako EM, Yako JM. A descriptive study of the reasons and consequences of pregnancy among single adolescent mothers in Lesotho. Curationis. 2007 Sep 28;30(3):74-81.
40. Atkins R, Gage G, Kelly TA, Joseph PV, Johnson S, Ojo K, Williams W. Exploring expressions of depression in Black single mothers. Issues in mental health nursing. 2018 Nov 2;39(11):935-45.
41. Katz R. Marital status and well-being: A comparison of widowed, divorced, and married mothers in Israel. Journal of divorce & remarriage. 1991 Jan 31;14(3-4):203-18.
42. Ritchie J. Social characteristics of a sample of solo mothers. The New Zealand Medical Journal. 1980 May 1;91(659):349-52.
43. Schlesinger B. Jewish female-headed one-parent families. Journal of divorce & remarriage. 1992 Mar 18;17(1-2):201-9.
44. Tcheng-Laroche F, Prince RH. Middle income, divorced female heads of families: their lifestyles, health and stress levels. Can. J. Psychiatry. 1979; 24:35-42.
45. Lee K, Vasileiou K, Barnett J. ‘Lonely within the mother’: An exploratory study of first-time mothers’ experiences of loneliness. Journal of health psychology. 2019 Sep;24(10):1334-44.
46. Au A, Chan YC, Cheung G, Yuen P, Lee T. Examining the correlation between parenting stress and social support and loneliness in Chinese mothers living in a socially impoverished community in Hong Kong. Journal of Psychology in Chinese Societies. 2008 Jul 1;9(2):167.
47. Berry JO, Jones WH. The parental stress scale: Initial psychometric evidence. Journal of Social and Personal Relationships. 1995 Aug;12(3):463-72.
48. Luthar SS, Ciciolla L. What it feels like to be a mother: Variations by children’s developmental stages. Developmental Psychology. 2016 Jan;52(1):143.
49. Hubert S, Aujoulat I. Parental burnout: When exhausted mothers open up. Frontiers in psychology. 2018 Jun 26;9:1021.
50. Oakley A, Rajan L, Turner H. Evaluating parent support initiatives: lessons from two case studies. Health & social care in the community. 1998 Sep;6(5):318-30.
51. Beck CT. The lived experience of postpartum depression: a phenomenological study. Nursing research. 1992 May;41(3):166–170.
52. Ugarriza DN. Postpartum depressed women's explanation of depression. Journal of Nursing Scholarship. 2002 Sep;34(3):227-33.
53. Badaru UM, Ogwumike OO, Adeniyi AF, Kaka B. Psychosocial adversities and depression in mothers of children with cerebral palsy in Nigeria. Journal of Pediatric Neurology. 2013 Jan 1;11(1):1-7.
54. Zaidi F, Nigam A, Anjum R, Agarwalla R. Postpartum depression in women: a risk factor analysis. Journal of Clinical and Diagnostic Research: Jcdr. 2017 Aug;11(8):QC13.
55. Luoma I, Korhonen M, Puura K, Salmelin RK. Maternal loneliness: concurrent and longitudinal associations with depressive symptoms and child adjustment. Psychology, health & medicine. 2019 Jul 3;24(6):667-79.
56. Luoma I, Korhonen M, Salmelin RK, Helminen M, Tamminen T. Long-term trajectories of maternal depressive symptoms and their antenatal predictors. Journal of Affective Disorders. 2015 Jan 1;170:30-8.
57. Junttila N, Ahlqvist‐Björkroth S, Aromaa M, Rautava P, Piha J, Räihä H. Intercorrelations and developmental pathways of mothers' and fathers' loneliness during pregnancy, infancy and toddlerhood–STEPS study. Scandinavian Journal of Psychology. 2015 Oct;56(5):482-8.
58. Lutz WJ, Hock E. Parental emotions following the birth of the first child: Gender differences in depressive symptoms. American Journal of Orthopsychiatry. 2002 Jul;72(3):415-21.
59. Valdez CR, Martinez E. Mexican immigrant fathers’ recognition of and coping with maternal depression: The influence of meaning-making on marital and co-parenting roles among men participating in a family intervention. Journal of Latinx Psychology. 2019 Apr 15.
60. Alvik A. Variables predicting low infant developmental scores: maternal age above 30 years is a main predictor. Scandinavian journal of public health. 2014 Mar;42(2):113-9.
61. Kruse JA, Williams RA, Seng JS. Considering a relational model for depression in women with postpartum depression. International journal of childbirth. 2014 Jan 1;4(3):151-68.
62. Al-Yagon M. Maternal personal resources and children’s socioemotional and behavioral adjustment. Child Psychiatry and Human Development. 2008 Sep 1;39(3):283.
63. Zafar N, Kausar R. Psychosocial Implications of Early Father Separation for Adolescents and their Mothers. Age. 2015 Jul 1;47:5-5.
64. Stednitz JN, Epkins CC. Girls' and mothers' social anxiety, social skills, and loneliness: Associations after accounting for depressive symptoms. Journal of Clinical Child and Adolescent Psychology. 2006 Feb 1;35(1):148-54.
65. Junttila N, Vauras M. Loneliness among school‐aged children and their parents. Scandinavian Journal of Psychology. 2009 Jun;50(3):211-9.
66. Henwood PG, Solano CH. Loneliness in young children and their parents. The Journal of genetic psychology. 1994 Mar 1;155(1):35-45.
67. Salo AE, Junttila N, Vauras M. Social and emotional loneliness: Longitudinal stability, interdependence, and intergenerational transmission among boys and girls. Family Relations. 2020 Feb;69(1):151-65.
68. Junttila N, Vauras M, Laakkonen E. The role of parenting self-efficacy in childrenś social and academic behavior. European journal of psychology of education. 2007 Mar 1;22(1):41-61.
69. Eni R, Phillips-Beck W, Mehta P. At the edges of embodiment: Determinants of breastfeeding for First Nations women. Breastfeeding Medicine. 2014 May 1;9(4):203-14.
70. Palmér L, Carlsson G, Brunt D, Nyström M. Existential security is a necessary condition for continued breastfeeding despite severe initial difficulties: a lifeworld hermeneutical study. International breastfeeding journal. 2015 Dec 1;10(1):17.
71. Milner JS, Wimberley RC. An inventory for the identification of child abusers. Journal of Clinical Psychology. 1979 Jan;35(1):95-100.
72. Vincenzi H, Grabosky F. Measuring the emotional/social aspects of loneliness and isolation. Journal of Social Behavior and Personality. 1987;2(2):257.
73. Medora NP, Wilson S, Larson JH. Attitudes toward parenting strategies, potential for child abuse, and parental satisfaction of ethnically diverse low-income US mothers. The Journal of Social Psychology. 2001 Jun 1;141(3):335-48.
74. Shahar G. Maternal personality and distress as predictors of child neglect. Journal of Research in Personality. 2001 Dec 1;35(4):537-45.
75. Kirkham MA, Schinke SP, Schilling RF, Meltzer NJ, Norelius KL. Cognitive-behavioral skills, social supports, and child abuse potential among mothers of handicapped children. Journal of Family Violence. 1986 Sep 1;1(3):235-45.
76. Gaudin JM, Polansky NA, Kilpatrick AC, Shilton P. Loneliness, depression, stress, and social supports in neglectful families. American journal of orthopsychiatry. 1993 Oct;63(4):597-605.
77. Milner JS, Wimberley AC. Prediction and explanation of child abuse. Journal of Clinical Psychology. 1980 Oct;36(4):875-84.
78. Polansky NA, Gaudin Jr JM, Ammons PW, Davis KB. The psychological ecology of the neglectful mother. Child Abuse & Neglect. 1985 Jan 1;9(2):265-75.
79. Polansky NA, Ammons PW, Gaudin Jr JM. Loneliness and isolation in child neglect. Social Casework. 1985 Jan;66(1):38-47.
80. Shapiro AF, Krysik J, Pennar AL. Who are the fathers in healthy families Arizona? An examination of father data in at‐risk families. American journal of orthopsychiatry. 2011 Jul;81(3):327.
81. Zare N, Ravanipour M, Bahreini M, Motamed N, Hatami G, Nemati H. Effect of a self-management empowerment program on anger and social isolation of mothers of children with cerebral palsy: A randomized controlled clinical trial. Evidence Based Care. 2017;7(3):35-44.
82. Richey CA, Lovell ML, Reid K. Interpersonal skill training to enhance social support among women at risk for child maltreatment. Children and Youth Services Review. 1991 Jan 1;13(1-2):41-59.
83. Dennis CL. The effect of peer support on postpartum depression: a pilot randomized controlled trial. The Canadian Journal of Psychiatry. 2003 Mar;48(2):115-24.
84. Dennis CL, Hodnett E, Kenton L, Weston J, Zupancic J, Stewart DE, Kiss A. Effect of peer support on prevention of postnatal depression among high risk women: multisite randomised controlled trial. Bmj. 2009 Jan 16;338:a3064.
85. Razani N, Kohn MA, Wells NM, Thompson D, Flores HH, Rutherford GW. Design and evaluation of a park prescription program for stress reduction and health promotion in low-income families: The Stay Healthy in Nature Everyday (SHINE) study protocol. Contemporary Clinical Trials. 2016 Nov 1;51:8-14.
86. Diehl K, Jansen C, Ishchanova K, Hilger-Kolb J. Loneliness at universities: determinants of emotional and social loneliness among students. International journal of environmental research and public health. 2018 Sep;15(9):1865.
87. Segel-Karpas D, Ayalon L, Lachman ME. Loneliness and depressive symptoms: The moderating role of the transition into retirement. Aging & mental health. 2018 Jan 2;22(1):135-40.
88. Hawkley LC, Cacioppo JT. Loneliness matters: A theoretical and empirical review of consequences and mechanisms. Annals of behavioral medicine. 2010 Oct 1;40(2):218-27.
89. Cacioppo JT, Hawkley LC, Thisted RA. Perceived social isolation makes me sad: 5-year cross-lagged analyses of loneliness and depressive symptomatology in the Chicago Health, Aging, and Social Relations Study. Psychology and aging. 2010 Jun;25(2):453.
90. Bauer A, Parsonage M, Knapp M, Iemmi V, Adelaja B. Costs of perinatal mental health problems. London: Centre for Mental Health; 2014
91. Kim HK, Capaldi DM, Pears KC, Kerr DC, Owen LD. Intergenerational transmission of internalising and externalising behaviours across three generations: Gender‐specific pathways. Criminal Behaviour and Mental Health. 2009 Apr;19(2):125-41.
92. Mason WA, Chmelka MB, Trudeau L, Spoth RL. Gender moderation of the intergenerational transmission and stability of depressive symptoms from early adolescence to early adulthood. Journal of youth and adolescence. 2017 Jan 1;46(1):248-60.
93. O’Connor JP, Alfrey L, Hall C, Burke G. Intergenerational understandings of personal, social and community assets for health. Health & place. 2019 May 1;57:218-27.
94. Jakobsen IS, Madsen LM, Mau M, Hjemdal O, Friborg O. The relationship between resilience and loneliness elucidated by a Danish version of the resilience scale for adults. BMC psychology. 2020 Dec;8(1):1-0.
95. Dennis CL. Peer support within a health care context: a concept analysis. International journal of nursing studies. 2003 Mar 1;40(3):321-32.
96. Thomson G, Balaam MC, Hymers K. Building social capital through breastfeeding peer support: insights from an evaluation of a voluntary breastfeeding peer support service in North-West England. International breastfeeding journal. 2015 Dec 1;10(1):15.
97. Qualter P, Brown SL, Rotenberg KJ, Vanhalst J, Harris RA, Goossens L, Bangee M, Munn P. Trajectories of loneliness during childhood and adolescence: Predictors and health outcomes. Journal of Adolescence. 2013 Dec 1;36(6):1283-93.
98. Mellor D, Stokes M, Firth L, Hayashi Y, Cummins R. Need for belonging, relationship satisfaction, loneliness, and life satisfaction. Personality and individual differences. 2008 Aug 1;45(3):213-8.
99. DiTommaso E, Spinner B. The development and initial validation of the Social and Emotional Loneliness Scale for Adults (SELSA). Personality and Individual Differences. 1993 Jan 1;14(1):127-34.
100. Ditommaso E, Brannen C, Best LA. Measurement and validity characteristics of the short version of the social and emotional loneliness scale for adults. Educational and Psychological Measurement. 2004 Feb;64(1):99-119.
101. Hoza B, Bukowski WM, Beery S. Assessing peer network and dyadic loneliness. Journal of Clinical Child Psychology. 2000 Feb 1;29(1):119-28.
102. Paloutzian RF, Ellison CW. Loneliness, spiritual well-being and the quality of life. Loneliness: A sourcebook of current theory, research and therapy. 1982:224-37.
103. Wittenberg MT (1986) Emotional and social loneliness: an examination of social skills, attributions, sex role and object relations perspectives. Unpublished doctoral dissertation, University of Rochester, New York
104. Asher SR, Wheeler VA. Loneliness and Social Dissatisfaction Questionnaire. Cambridge University Press; 1985.
105. Aching MC, Granato TM. The good enough mother under social vulnerability conditions. Estudos de Psicologia (Campinas). 2016 Mar;33(1):15-24.
106. Ayers S, Crawley R, Webb R, Button S, Thornton A, HABiT collaborative group, Smith H, Bradley R, Lee S, Moore D, Field A. What are women stressed about after birth?. Birth. 2019 Dec;46(4):678-85.
107. Björk M, Wiebe T, Hallström I. Striving to survive: Families’ lived experiences when a child is diagnosed with cancer. Journal of Pediatric Oncology Nursing. 2005 Sep;22(5):265-75.
108. Bloom T, Glass N, Curry MA, Hernandez R, Houck G. Maternal stress exposures, reactions, and priorities for stress reduction among low‐income, urban women. Journal of midwifery & women's health. 2013 Mar;58(2):167-74.
109. Boman KK, Viksten J, Kogner P, Samuelsson U. Serious illness in childhood: the different threats of cancer and diabetes from a parent perspective. The Journal of pediatrics. 2004 Sep 1;145(3):373-9.
110. Boxill NA. " How Would You Feel...?": Clinical Interviews With Black Adolescent Mothers. Child & Youth Services. 1987 Jun 12;9(1):41-51.
111. Cameron G. Motivation to join and benefits from participation in parent mutual aid organizations. Child welfare. 2002 Jan 1;81(1).
112. Chan YC, LAM GL, Kwok SM. Evaluation study on a home visiting program in Hong Kong. Asia Pacific Journal of Social Work and Development. 2005 Dec 1;15(2):41-54.
113. Cronin C, McCarthy G. First-time mothers-identifying their needs, perceptions and experiences. Journal of clinical nursing. 2003 Mar 1;12(2):260-7.
114. Cutrona CE. Objective determinants of perceived social support. Journal of personality and social psychology. 1986 Feb;50(2):349.
115. Dykstra P, Keizer R. The wellbeing of childless men and fathers in mid-life. Ageing and society. 2009 Nov 1;29(8):1227-42.
116. Ekas NV, Pruitt MM, McKay E. Hope, social relations, and depressive symptoms in mothers of children with autism spectrum disorder. Research in Autism Spectrum Disorders. 2016 Sep 1;29:8-18.
117. Eni R, Phillips-Beck W, Mehta P. At the edges of embodiment: Determinants of breastfeeding for First Nations women. Breastfeeding Medicine. 2014 May 1;9(4):203-14.
118. Ergün S, Ertem G. Difficulties of mothers living with mentally disabled children.
119. Essex EL, Petras D, Massat CR. Predictors of loneliness among court-involved and substance abusing mothers. Women & Criminal Justice. 2006 Jan 1;17(2-3):63-74.
120. Fabian HM, Rådestad IJ, Waldenström U. Characteristics of primiparous women who are not reached by parental education classes after childbirth in Sweden. Acta Paediatrica. 2006 Nov;95(11):1360-9.
121. Forinder U, Norberg AL. “Now we have to cope with the rest of our lives”. Existential issues related to parenting a child surviving a brain tumour. Supportive care in cancer. 2010 May 1;18(5):543-51.
122. Farzizadeh Z. A Study of the Effects of Drug Addicted Fathers on Families in Tehran, Iran. Pertanika Journal of Social Sciences & Humanities. 2018 Feb 2.
123. Gladow NW, Ray MP. The impact of informal support systems on the well being of low income single parents. Family Relations. 1986 Jan 1:113-23.
124. Gosztyla T, Prokopiak A. Sense of loneliness in parents of children with autism spectrum disorder and children with intellectual disability. Psychoterapira. 2017 Jan 1(3):81-92.
125. Gosztyła, T. and Prokopiak, A., 2019. Mediating role of lack of support for the relationship between extraversion and sense of loneliness in parents of children with autism spectrum disorder and parents of children with intellectual disability. Journal of Psychopathology. 2019, 25:51-57.
126. Gove WR, Geerken MR. The effect of children and employment on the mental health of married men and women. Social Forces. 1977 Sep 1;56(1):66-76.
127. Graffigna G, Bosio C, Cecchini I. Assisting a child with tuberous sclerosis complex (TSC): a qualitative deep analysis of parents’ experience and caring needs. BMJ open. 2013 Dec 1;3(12).
128. Grootenhuis MA, Last BF. Predictors of parental emotional adjustment to childhood cancer. Psycho‐Oncology: Journal of the Psychological, Social and Behavioral Dimensions of Cancer. 1997 Jun;6(2):115-28.
129. Gulhati A, Minty B. Parental health attitudes, illnesses and supports and the referral of children to medical specialists. Child: care, health and development. 1998 Jul;24(4):295-315.
130. Halford WK, Sweeper S. Trajectories of adjustment to couple relationship separation. Family process. 2013 Jun;52(2):228-43.
131. Halsa A. Trapped between madness and motherhood: Mothering alone. Social Work in Mental Health. 2018 Jan 2;16(1):46-61.
132. Hamama-Raz Y, Hamama L. Quality of life among parents of children with epilepsy: a preliminary research study. Epilepsy & Behavior. 2015 Apr 1;45:271-6.
133. Hudson DB, Campbell-Grossman C, Kupzyk KA, Brown SE, Yates B, Hanna KM. Social support and psychosocial well-being among low-income, adolescent, African American, first-time mothers. Clinical nurse specialist CNS. 2016 May;30(3):150.
134. Hudson DB, Elek SM, Campbell-Grossman C. Depression, self-esteem, loneliness, and social support among adolescent mothers participating in the new parents project. Adolescence. 2000 Sep 22;35(139):445-153.
135. Hudson DB, Campbell-Grossman C, Hertzog M. Effects of an internet intervention on mothers’ psychological, parenting, and health care utilization outcomes. Issues in comprehensive pediatric nursing. 2012 Jul 1;35(3-4):176-93.
136. Igarashi Y, Horiuchi S, Porter SE. Immigrants’ experiences of maternity care in Japan. Journal of community health. 2013 Aug 1;38(4):781-90.
137. Khan S, Ion A, Alyass A, Greene S, Kwaramba G, Smith S, Carvalhal A, Kennedy VL, Walmsley S, Loutfy M. Loneliness and perceived social support in pregnancy and early postpartum of mothers living with HIV in Ontario, Canada. AIDS care. 2019 Mar 4;31(3):318-25.
138. Kim HK, Kim MK. Mothers' Survival Experiences in Cases of Incestuous Sexual Abuse of Girls 1. Asian Journal of Women's Studies. 2011 Jan 1;17(2):96-119.
139. Klajmon-Lech U. Early support and early intervention as a support for the child and their family based on the biographies of parents of children with rare genetic diseases. Psychiatria i Psychologia Kliniczna. 2016;16(4).
140. Kulkarni SJ. The relational consequences of interpersonal violence (IPV) for adolescent mothers. Youth & Society. 2009 Sep;41(1):100-23.
141. Layton S. Left alone to hold the baby. Infant Observation. 2007 Dec 1;10(3):253-65.
142. LeDrew HM, Moores P, Read T, O'Regan-Hogan M. He's here and he's gone; he's here and he's gone... The experiences of new mothers in rural Newfoundland and Labrador, Canada, whose partners work away from home. Rural and remote health. 2018;18(3):5.
143. Maurice‐Stam H, Oort FJ, Last BF, Grootenhuis MA. Emotional functioning of parents of children with cancer: the first five years of continuous remission after the end of treatment. Psycho‐Oncology: Journal of the Psychological, Social and Behavioral Dimensions of Cancer. 2008 May;17(5):448-59.
144. Mayers AM, Naples NA, Nilsen RD. Existential issues and coping: A qualitative study of low-income women with HIV. Psychology & Health. 2005 Feb 1;20(1):93-113.
145. Nahas V, Amasheh N. Culture care meanings and experiences of postpartum depression among Jordanian Australian women: A transcultural study. Journal of Transcultural Nursing. 1999 Jan;10(1):37-45.
146. Nyström K, Öhrling K. Parental support: mothers' experience of electronic encounters. Journal of Telemedicine and Telecare. 2006 Jun 1;12(4):194-7.
147. Omer-Salim A, Suri S, Dadhich JP, Faridi MM, Olsson P. Theory and social practice of agency in combining breastfeeding and employment: A qualitative study among health workers in New Delhi, India. Women and Birth. 2014 Dec 1;27(4):298-306.
148. Paris R, Dubus N. Staying connected while nurturing an infant: A challenge of new motherhood. Family Relations. 2005 Jan;54(1):72-83.
149. Richman N. The effects of housing on pre‐school children and their mothers. Developmental Medicine & Child Neurology. 1974 Feb;16(1):53-8.
150. Rolls C, Hanna B. What About the Mother and Family when an Infant Doesn't Sleep?. Australian Journal of Primary Health. 2001;7(3):49-53.
151. Rozenzweig M, Gampel J, Dasberg H. Separation and stress in the military family in Israel. Israel Journal of Psychiatry and Related Sciences. 1981;18(2): 87-98.
152. Shorey S, Chee C, Chong YS, Ng ED, Lau Y, Dennis CL. Evaluation of technology-based peer support intervention program for preventing postnatal depression: Protocol for a randomized controlled trial. JMIR research protocols. 2018;7(3):e81.
153. Silverstein M, Reid S, DePeau K, Lamberto J, Beardslee W. Functional interpretations of sadness, stress and demoralization among an urban population of low-income mothers. Maternal and child health journal. 2010 Mar 1;14(2):245-53.
154. Skar AM, von Tetzchner S, Clucas C, Sherr L. The long-term effectiveness of the International Child Development Programme (ICDP) implemented as a community-wide parenting programme. European Journal of Developmental Psychology. 2015 Jan 2;12(1):54-68.
155. Sorenson DS. Healing traumatizing provider interactions among women through short-term group therapy. Archives of psychiatric nursing. 2003 Dec 1;17(6):259-69.
156. Stewart M, Dennis CL, Kariwo M, Kushner KE, Letourneau N, Makumbe K, Makwarimba E, Shizha E. Challenges faced by refugee new parents from Africa in Canada. Journal of immigrant and minority health. 2015 Aug 1;17(4):1146-56.
157. Stewart M, Makwarimba E, Letourneau NL, Kushner KE, Spitzer DL, Dennis CL, Shizha E. Impacts of a Support Intervention for Zimbabwean and Sudanese Refugee Parents:" I Am Not Alone". Canadian Journal of Nursing Research Archive. 2015 Dec 15;47(4).
158. Tirgari B, Rayyani M, Cheraghi MA, Mangeli M. Experiences of iranian Teen mothers with parenting stress: a qualitative study. Comprehensive child and adolescent nursing. 2020 Jul 2;43(3):203-16.
159. Tuominen M, Junttila N, Ahonen P, Rautava P. The effect of relational continuity of care in maternity and child health clinics on parenting self‐efficacy of mothers and fathers with loneliness and depressive symptoms. Scandinavian journal of psychology. 2016 Jun;57(3):193-200.
160. White AE, McLennan JP. Single Parents and Social Support Networks: The Contribution of ‘Parents Without Partners’. Australian Journal of Sex, Marriage and Family. 1987 Nov 1;8(4):164-72.
161. Yárnoz S, Plazaola M, Etxeberria J. Adaptation to divorce: An attachment-based intervention with long-term divorced parents. Journal of Divorce & Remarriage. 2008 Sep 24;49(3-4):291-307.
162. Zekeri AA. Livelihood strategies of food-insecure poor, female-headed families in rural Alabama. Psychological reports. 2007 Dec;101(3_suppl):1031-6.
